# New Approach in Ulcer Prevention and Wound Healing Treatment using Doxycycline and Amoxicillin/LDH Nanocomposites

**DOI:** 10.1038/s41598-019-42842-2

**Published:** 2019-04-23

**Authors:** Fatma I. Abo El-Ela, Ahmed A. Farghali, Rehab K. Mahmoud, Nada A. Mohamed, S. A. Abdel Moaty

**Affiliations:** 1 Lecturer of Pharmacology, Department of Pharmacology, Faculty of Veterinary Medicine, Beni-Suef University, Beni-Suef, Egypt; 2 Materials Science and Nanotechnology Department, Faculty of Postgraduate Studies for Sciences, Beni-Suef University, Beni-Suef, Egypt; 3 Department of Chemistry, Faculty of Science, Beni-Suef University, Beni-Suef, Egypt; 4 Materials Science Lab, Department of Chemistry, Faculty of Science, Beni-Suef University, Beni-Suef, Egypt

**Keywords:** Biogeochemistry, Bioinorganic chemistry

## Abstract

Doxycycline (DOX) and amoxicillin (AMOX) are important Broad-spectrum antibiotics used in treating multiple human and animal diseases. For the sake of exploring novel medical applications, both antibiotics were loaded into magnesium aluminium layer double hydroxide (Mg-Al)/LDH nanocomposite through the co-precipitation method. The synthesized materials were characterized by XRD, FT-IR, particle size analysis, FESEM and HRTEM. Acute toxicological studies were conducted using median lethal dose LD_50_, where a total number of 98 rats (200–150 gm) of both sexes were used. An experimental wound was aseptically incised on the anterior-dorsal side of each rat, while 98% of pure medical ethanol was used for ulcer induction. Acute toxicity, wound closure rate, healing percentages, ulcer index, protective rate and histopathological studies were investigated. Antibiotic Nanocomposites has significantly prevented ulcer formation and improved wound healing process to take shorter time than that of the typical processes, when compared with that of same drugs in microscale systems or commercial standard drugs. These results were confirmed by the histopathological findings. By converting it into the Nanoform, which is extremely important, especially with commonly used antibiotics, novel pharmacological properties were acquired from the antibiotics. The safe uses of DOX/LDH and AMOX/LDH Nanocomposites in this study were approved for biomedical applications.

## Introduction

For complete recovery, the recuperation of wounds in individual hosts involves various biological processes. Cellular and immunological entrapment into the wound is commonly categorized into three main phases for wound restoration^1^ (inflammation, proliferation and remodelling). Chronic wounds are recognized by the deterioration in their wound healing process leading to putrefaction, infection or even resection^2^. The typical properties of the wound treating material includes having an anti-infective effect^3^, therefore, it is intended to use antimicrobial agents loaded onto nanomaterials for enhancing the wound recovery rate with controlled release and high penetration power.

The main cause of gastrointestinal ulcers and infections for both adults and children^4^ worldwide is the *Helicobacter pylori* bacterial infection. The preferred spot for *H*. *pylori* bacteria is the gastric mucous lining adherent to the gastric epithelium, as normal antimicrobials are unable to reach this spot for treatment which in turn makes its treatment a quite complex process^5^. Besides, the used antimicrobials cannot reach the infected spot in a sufficient effective concentration or in an active form^6^. Unlike Nano antibiotics characterized by its higher penetrating power and its ability to reach the *H*. *pylori* bacteria existing in the epithelial lining.

Disadvantages of antibiotics such as overdoses, toxicity, resistance, stability and solubility can all be controlled via using nanomaterials in Drug delivery. Reformulations of antibiotics using nanoparticles or nanocomposites have greater advantages, with regard to its gastrointestinal adhesion to the mucous or wound area penetration power, due to its small size. They give a superior activity against the *H*. *pylori* bacteria^7^. The chosen loaded antibiotic has an important impact as it is effective either against *H*. *pylori* bacteria for ulcer treatment or the other bacteria infecting wounds. Doxycycline broad-spectrum antibiotic of the Oxtetracycline class is considered the most effective one against *H*. *pylori* bacterial infection^8^.

Due to its stability and anion exchange properties in physiological media, the Layered double hydroxides (LDHs) or hydrotalcite-like compounds are considered one of the most common molecular nanomaterials used in drug delivery^9^. Anionic clays or LDHs have a general formula of [M(II)(1−x)M(III)x(OH)_2_](Ax/n)·mH_2_O, where M(II) is a divalent cation such as Mg, Ni, Zn, Cu or Co and M(III) is a trivalent cation such as Al, Cr, Fe or Ga, while An− is an anion such as CO_3_^−^, Cl^−^, NO_3_^−^ or organic anions^10^.

Doxycycline is a FDA approved broad spectrum bacteriostatic drug which acts by inhibiting the protein synthesis^11^. It might be taken together with a sub-therapeutic dosage and might require administering multiple dosing for a long period^12^. Therefore, the localization of this drug will increase its efficacy and consequently decrease the repeated dosage administration.

Amoxicillin, which is a broad-spectrum bactericidal safe and cheap antibiotic of the penicillin family group, has a high therapeutic index. It enters the body through multiple routes and its essential drug is approved by WHO (World Health Organization). However; Amoxicillin is of a short half-life (1h) and it is unstable in the aqueous media^13^, so the aim of this work is to increase the Amoxicillin’s efficacy and stability besides investigating its effect on wound healing and ulcer prevention processes.

To complete our work on a series of LDHs^14–17^ and due to the use of Mg-Al in a wide range of biomedical application; we therefore designed this bio composite in our piece of work whereas our aim is to synthesize and characterize Mg-Al/LDH Doxycycline and Mg-Al/LDH Amoxicillin composites in addition to exploring novel applications for the antibiotics use. We are establishing an excision of wound and ulcer model for integrating the semi-quantitative and quantitative appreciation of selected parameters for wound and ulcer evaluation, besides exploring the potential applications for doxycycline and amoxicillin with LDH nanocomposites. Additionally we attempted to increase the Amox. efficiency & stability by investigating the potential application of DOX and AMOX loaded on LDH as Nanocomposites.

## Results and Discussion

**Figure 1:**
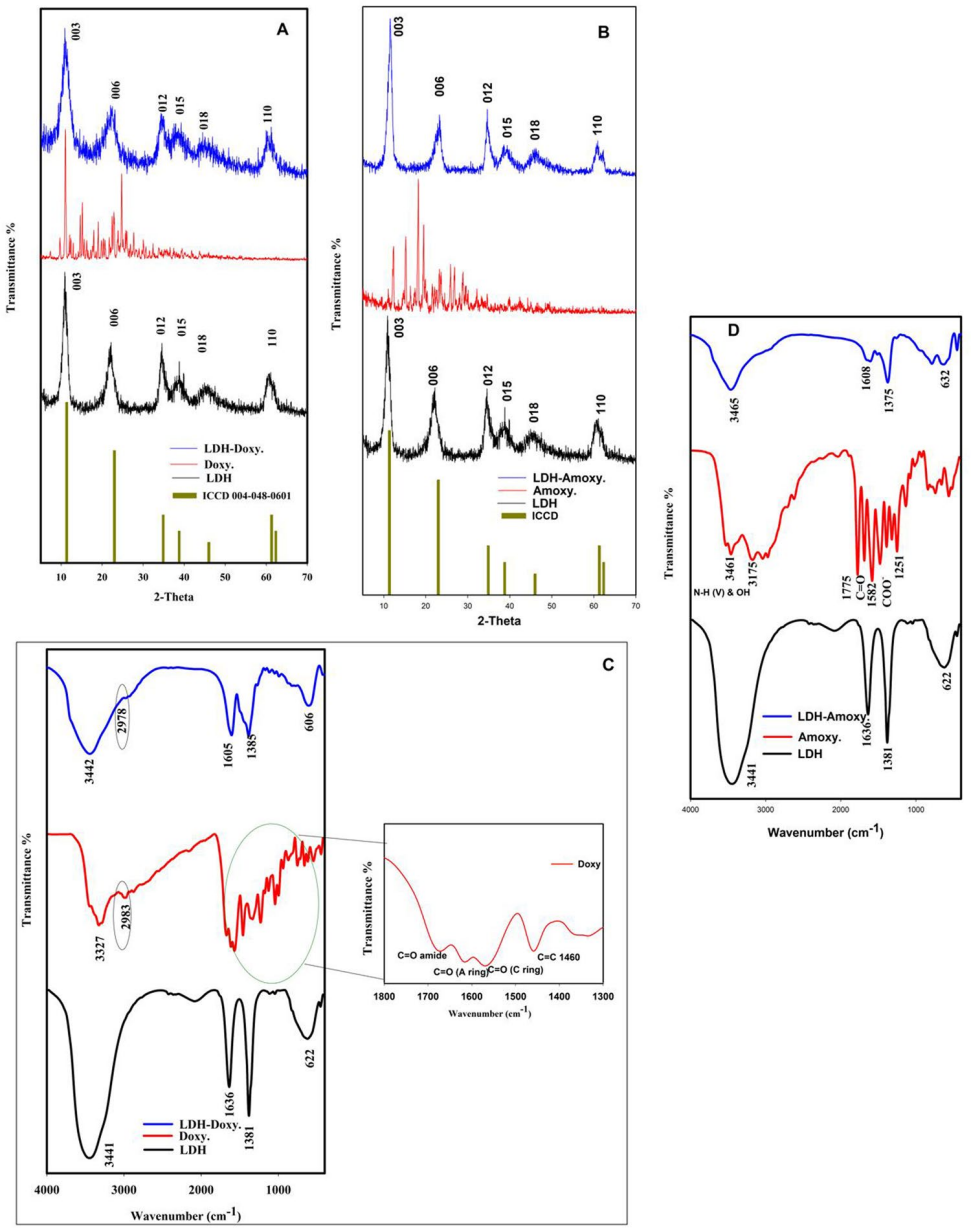
(**A**) XRD patterns of ICCD, Mg-Al/LDH, Doxycycline and Doxycycline/LDH. (**B**) ICCD card no (00-048-0601), Mg-Al/LDH, Amoxicillin and Amoxicillin-LDH. (**C**) FTIR spectra of Mg Al LDH, Doxycycline and Doxycycline -LDH. (**D**) FTIR spectra of Mg Al LDH, Amoxicillin and Amoxicillin -LDH.

Figure 1A shows the XRD patterns of LDH, doxycycline and doxycycline/LDH. The XRD results confirm the structure of the LDH relative to ICDD card no (00-048-0601)^18^. The average crystallite size (L) was calculated using the Debye–Scherer formula, L = kλ/Bcosθ, where k is the shape factor, B is the spectral line width at half maximum intensity, λ is the X-ray wavelength and θ is the Bragg diffraction angle. The Mg-Al LDHs were indexed to a rhombohedral crystal structure with a space group of R-3m and unit cell parameters a = b = 3.04 Å and c = 23.5 Å. The basal planes (003) and (006) as well as the broadening of the (015) and (018) planes confirmed the layered structure of the LDHs. The crystallite size was 16.441 nm. All peaks of doxycycline/LDH are similar to those of the diffraction pattern of the LDHs, confirming the stability of the structure after loading. Figure 1B shows that all patterns indicate the formation of a well-crystallized hydrotalcite-like phase of amoxicillin–LDH. Interestingly, the basal spacing gaps in the case of DOX/LDH and AMOX/LDH were 0.112 and 0.419 Å, respectively, after loading with DOX and AMOX. These results suggest that the change in the d spacing was very small; indicating that DOX or AMOX exhibits less effective penetration into the Mg-Al/LDH interlayer^19^. Therefore, the change in the XRD pattern provides indirect evidence that the drugs were are adsorbed on the surface of the positive LDH. No material has been contaminated by atmospheric CO_2_, which was also confirmed by the EDAX.

The FT-IR spectrum was investigated to estimate the structure of the Mg-Al/LDH, amoxicillin/LDH and doxycycline/LDH nanocomposites, as shown in Fig. 1(C,D). The chemical bonds in the Mg-Al-NO_3_ LDH were identified by the band centered at 622 cm^−1^ and attributed to M–O–M vibration^20,21^. This band, similarly to that associated with M–O–H bending^21^, involves the translational motion of oxygen metal ions in brucite-like layers^22,23^. The strong broad band at 3441 cm^−1^ is related to the stretching vibrations of the H-bond of the OH group (ν O–H) in brucite-like layers^21^. The bending vibration (δ H_2_O) of H_2_O molecules in the interlayers^20^ appeared at 1638 cm^−1^. The peak located at 1381 cm^−1^ is related to the ν3 stretching vibration of the NO_3_ groups in the LDH interlayer.

Interestingly, Fig. 1C,D shows the presence of important bands of AMOX and DOXY in the spectra of Mg-Al LDH/AMOX and Mg-Al LDH/DOXY composites, indicating the successful loading of drugs in the Mg-Al LDH host^24,25^. In addition, the intensity of the NO_3_ peak at 1381 cm^−1^ decreased, indicating the exchange of nitrate anions by the antibiotics^24^. In addition, some peaks shifted to new values; for example, the NO_3_ group peak at 1381 cm^−1^ shifted to 1375 cm^−1^, while some peaks, such as the peak related to the carbonyl group C=O stretching vibration of COO^−^ in AMOX at 1775 cm^−1^, disappeared for the Mg-Al LDH/AMOX nanocomposite, confirming the loading process.

**Figure 2:**
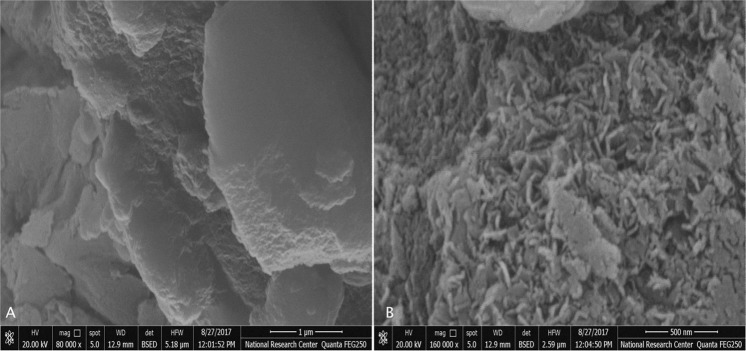
(**A**,**B**) FESEM of Mg-Al LDH.

The morphology of the Mg-Al/LDH was investigated using FESEM images, which supported the results of the XRD; where all layers are clustered in a plate-like morphology, as shown in Fig. 2. This is due to the slowness of the homogeneity and nucleation processes.

**Figure 3:**
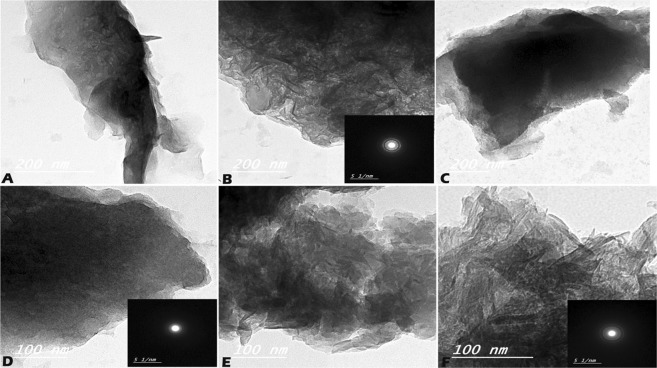
HRTEM of (**A**,**B**) Mg-Al LDH, (**C**,**D**) Doxycycline –LDH, and (**E**,**F**) Amoxicillin–LDH.

The HRTEM micrographs presented further confirmation on the observations of XRD, In Fig. 3A,B, the HRTEM images showed the layered structures Mg-Al/LDH. While in Fig. 3C,D show Mg-Al/LDH after loading Doxycycline and finally Fig. 3E,F show Amoxicillin drugs on Mg-Al/LDH. The layered structure appeared, therefore the LDH structure is stable after drug loading. The Selected Area Electron Diffraction (SAED) pattern assumed the good crystallinity of LDHs.

The Z-average diameters and potentials of Mg-Al/LDH, doxycycline/LDH and amoxicillin/LDH at different pH values, as determined by dynamic light scattering, are listed in Table 1. The diameters of doxycycline/LDH and amoxicillin/LDH were 723.1 and 1217.8 nm, respectively, larger than that of Mg-Al/LDH. This discrepancy could be attributed to the adsorption of amoxicillin and doxycycline on the surface of LDH. The optimized zeta potentials of Mg-Al/LDH, doxycycline/LDH and amoxicillin/LDH at different pH values are shown in Table 1. The positive zeta potential of Mg-Al/LDH at different pH values is attributed to the nature of the LDH. The zeta potential decreased for AMOX/LDH and DOX/LDH compared with that of pure LDH. It reflected that AMOX and DOX were extensively successful nanocomposites formulations^26^

**Table 1:** Zeta potential and particle size of Mg-Al LDH values at different pH for Doxycycline-LDH and Amoxicillin-LDH.

pH		Zeta potential (mV)	Z-average size (nm)
2.66	Mg-Al LDH	33.70 ± 6.57	291.7
3.75		27.80 ± 4.96	277.3
6.20		30.40 ± 4.07	395.9
9.20		29.00 ± 4.07	301.2
10.60		21.50 ± 4.11	2539.0
4.00	Doxycycline/-Al LDH	−7.760 ± 6.03	723.1
4.00	Amoxicillin/Mg-Al LDH	2.420 ± 5.53	1217.8

Data in Tables (2 and 3) present the results of the acute toxicity studies of DOX/LDH and AMOX/LDH nanocomposite in mice after the oral administration. Symptoms of toxicity were observed following DOX/LDH and AMOX/LDH nanocomposites administration in diseases like tremors, rapid respiration, arched back, convulsions and coma followed by death. In DOX/LDH Nanocomposites, mortality probabilities were found to initiate at 600 mg/kg b.wt. compared to 1100 mg/kg b.wt. in AMOX/LDH. The LD_50_ was calculated to be 1100 and 1210 mg/kg b.wt. in DOX/LDH and AMOX/LDH respectively and 100% mortality (LD_100_) was achieved at dose of 1600 and 1500 mg/kg b.wt. in both Nanocomposites respectively. These results indicated the safe use of both DOX/LDH and AMOX/LDH Nanocomposites in pharmacological studies.

**Table 2:** Determination the LD_50_ of DOX/LDH after oral administration in mice (n = 5).

Group	Dose (mg/kg b.wt.	No. of Animals/group	No. of dead Animals	A	B	A × B	Σ (A × B)
1	200	5	0	200	0	0	
2	400	5	0	200	0.5	100	
3	600	5	1	200	1	200	
4	800	5	1	200	1.5	300	
5	1000	5	2	200	2	400	
6	1200	5	2	200	3	600	
7	1400	5	4	200	4.5	900	
8	1600	5	5	200	—	—	2500

*LD*50 = *largest dose cause* 100% *deaths* $-\frac{\Sigma \,(A\times B)}{N}$ = 1600 − 2500/5 = 1100 mg/kg.b wt.

“Minimum lethal dose of DOX/LDH is 1100 mg/kg b.wt.”

**Table 3:** Determination the LD_50_ of AMOX/LDH after oral administration in mice (n = 5).

Group	Dose (mg/kg b.wt.	No. of Animals/group	No. of dead Animals	A	B	A × B	Σ (A × B)
1	800	5	0	100	0.5	50	
2	900	5	1	100	1	100	
3	1000	5	1	100	1	100	
4	1100	5	1	100	1.5	150	
5	1200	5	2	100	2.5	250	
6	1300	5	3	100	3.5	350	
7	1400	5	4	100	4.5	450	
8	1500	5	5	100	—	—	1450

*LD*50 = *largest dose cause* 100% *deaths* $-\frac{\Sigma (A\times B)}{N}$ = 1500 − 1450/5 = 1210 mg/kg.b wt.

“Minimum lethal dose of AMOX/LDH is 1210 mg/kg b.wt.”

We considered LD_50_ values of 1/200 and 1/30 for the DOX/LDH nanocomposite and the AMOX/LDH nanocomposite, respectively, to evaluate the ulcer treatment in this study. Toxicological studies for both nanocomposites had been investigated in mice and the wound and ulcer applications had been investigated in rats as in our study no significant difference observed between the LD_50_ between the rats and mice. In many studies the therapeutic dose in rats is almost equivalent to that dose in mice, but if you ask about the toxic dose it varies in significant aspects. If you establish an acute toxicity study to settle a new and safest therapeutic dose using mice you can certainly sure that in that dose is safe enough for rat. For selecting the perfect therapeutic dose in rats compared to mice you need to adjust a few (e.g. only +/−5%) dosage regimen only in some cases or not differs. So, dose adjustment in mice is suggested and then you can easily convert and adjust it in rats, which as well as ensures economic benefit too as previously mentioned in other studies^27–34^ (1–7).

According to the LD_50_ and with increasing drug dose, the toxicity increased in the study, as shown in Tables 2 and 3. In addition, we chose these doses at first for a pilot study, and calculated the LD_50_ based on the results obtained. Furthermore, we found the same dose for both amoxicillin and doxycycline based on in previous literature^35,36^. The dosage of the active drug component (only AMOX or DOX) was as follows: amoxicillin-loaded NPs or amoxicillin powder was orally administered to the SD rats (250–300 g, six rats for each group) fasted for 8 h at an amoxicillin dose of 40 mg/kg^35^. The doxycycline dose was selected according to the LD_50_ and according to a previous study that reported that doxycycline has a pH of approximately 3.3 and a dose of 5 mg/kg b.wt.^36^.

**Figure 4:**
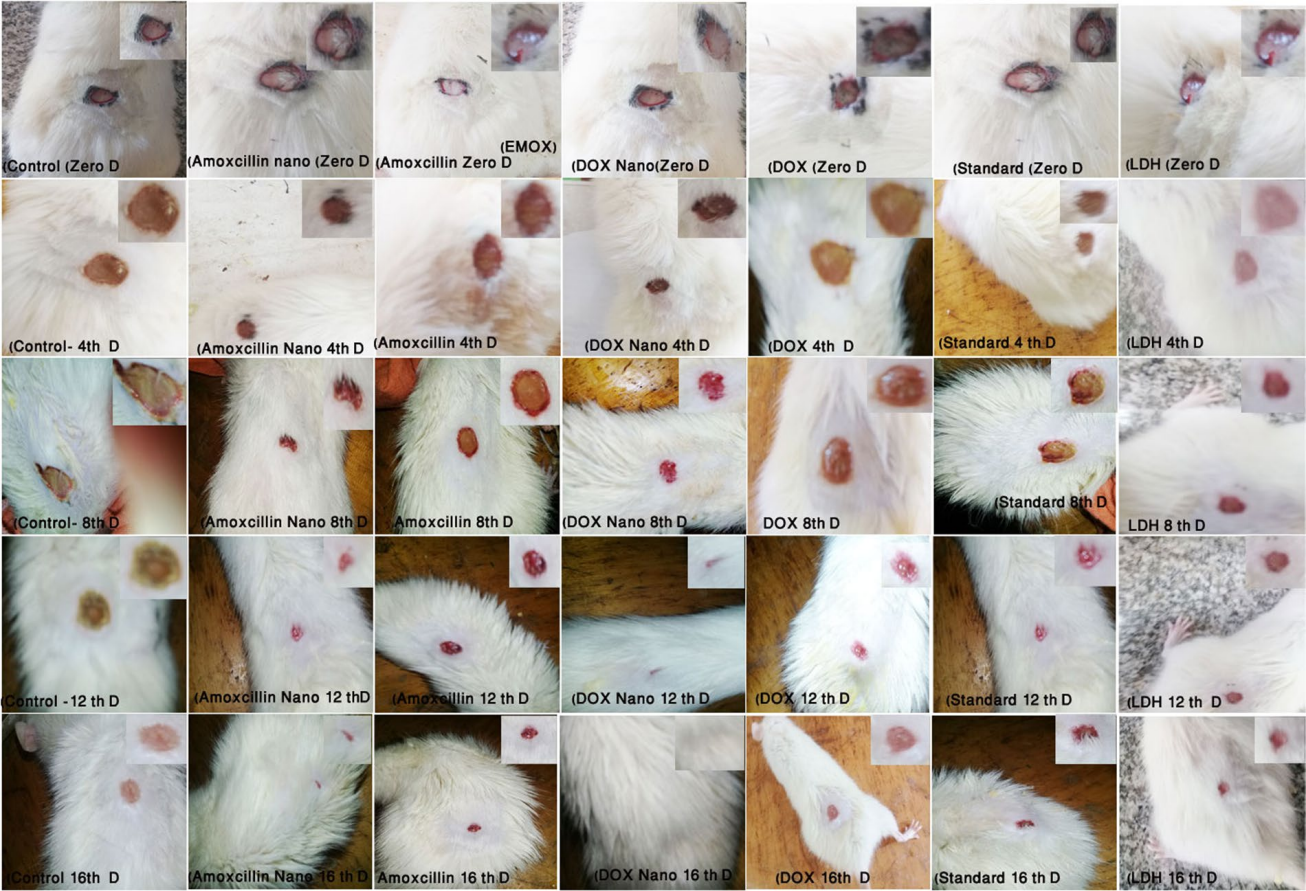
Wound healing activity of G1 (CNT), G2 (AMOX/LDH), G3 (AMOX), G4 (DOX/LDH), G5 (DOX), G6 (standard), and G7 (Mg AL) in rats (n = 6), at Zero, 4, 8, 12 and 16 days of treatment.

Rats treated with topical application of DOX/LDH (G4) and AMOX/LDH (G2) nanocomposites ointment showed a significant increase in the wound healing percentages and in the wound closure size at 4 *(p* < *0*.*001*, *p* < *0*.*00)*, 8 *(p* < *0*.*001*, *p* < *0*.*001)*,12 *(p* < *0*.*001*, *p* < *0*.*00)* and 16 day *(p* < *0*.*001*, *p* < *0*.*001)* of topical administration on the wound area in DOX/LDH and AMOX/LDH respectively when compared to non-treated control group. In addition, a significant increase in the wound healing activity was revealed for G3 (AMOX), G5 (DOX), G6 (standard) and G7 (Mg AL) at 4 (*p* < *0*.*5*, *p* < *0*.*001*, *p* < *0*.*001*, *p* < *0*.*5*), 8 (*p* < *0*.*001*), 12 (*p* < *0*.*001*) and 16 days (*p* < *0*.*001*) of treatment respectively in comparison to the non-treated control groups. Non-significant variation was observed between G3, G5, G6 and G7. Significant increase in the wound closure rate is shown in G2 and G4 as compared to other groups other than control G3, G5, G6 and G7 (Table 4), (Fig. 4) and (Chart 1). Complete wound healing was achieved after 18 days in DOX/LDH (G4) and AMOX/LDH (G2) nanocomposites and G6 (standard), compared to 24 days for complete closure in other groups; indicating a faster highly efficient wound healing process in the nanocomposites with anti-microbial drugs.

**Table 4:** Wound healing activity of DOX/LDH and AMOX/LDH Nano composite in rats. (n = 6).

Group	Wound size (mm) and Percentage of wound healing at			
	4^th^ day	8^th^ day	12^th^ day	16^th^ day
G1	100.00 ± 0.00^a^	100.00 ± 0.00^a^	66.00 ± 1.5^a^	56.33 ± 4.3^a^
Control	0%	0%	34%	44%
G2	60.00 ± 1.8^b^	42.66 ± 1.6^c^	20.00 ± 1.3	5.66 ± 1.4^a^
AMOX/LDH	40%	57.34%	80%	94.34%
G3	81.00 ± 0.9^c^	58.66 ± 2.6^cb^	24.66 ± 0.6	9.33 ± 2.6^c^
AMOX	19%	41.34%	75.34%	90.67%
G4	41.33 ± 4.9	29.53 ± 4.8	16.00 ± 1.3	4.33 ± 2.4
DOX/LDH	58.67%	70.47%	84%	95.67%
G5	72.00 ± 0.00^bc^	25.00 ± 2.00	14.66 ± 1.3	14.66 ± 3.1^c^
DOX	28%	75%	85.34%	85.34%
G6	72.00 ± 4.6^bc^	59.00 ± 2.6^cb^	10.66 ± 1.3	10.66 ± 1.3^c^
Standard	28%	41%	89.34%	89.34%
G7	78.04 ± 3.00^c^	72.33 ± 2.8^b^	17.66 ± 1.4	17.66 ± 1.4^bc^
Mg-Al	21.96%	27.67%	82.34%	82.34%

Means within the same column having the same or no manuscript denote non-significant variation *(P* < *0*.*05*).

**Figure 5:**
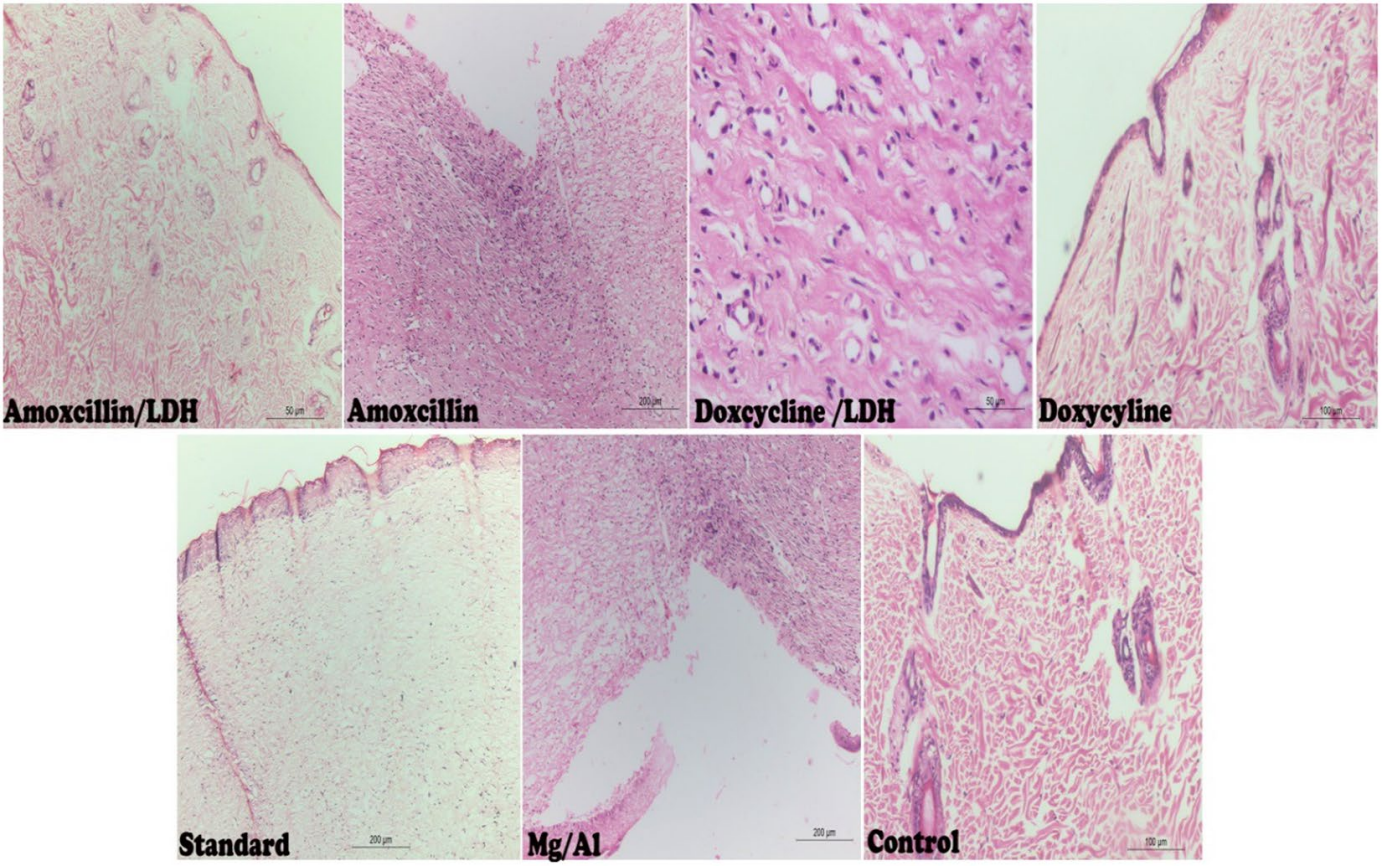
Histopathological investigation of the different treatments on Skin wound showed complete epithelial healing with normal vasculature and cellular infiltration. Amoxicillin and Doxycycline groups showed complete epithelial healing with congestion while standard group reveal normal epithelial forming on the other hand control group and Mg/Al showed severe damage on the epithelial surface.

Histopathological investigations for the skin specimens of the different treatments revealed that both AMOX/LDH and DOX/LDH nanocomposites led to complete wound healing with normal epithelial forming and vasculature. Moreover, the same healing activity appeared in a standard group but of lower efficiency than these two groups. Other groups of AMOX, DOX, Mg/Al and control groups, on the other hand, showed complete wound healing with congestion and epithelial rupture (Fig. 5).

**Figure 6:**
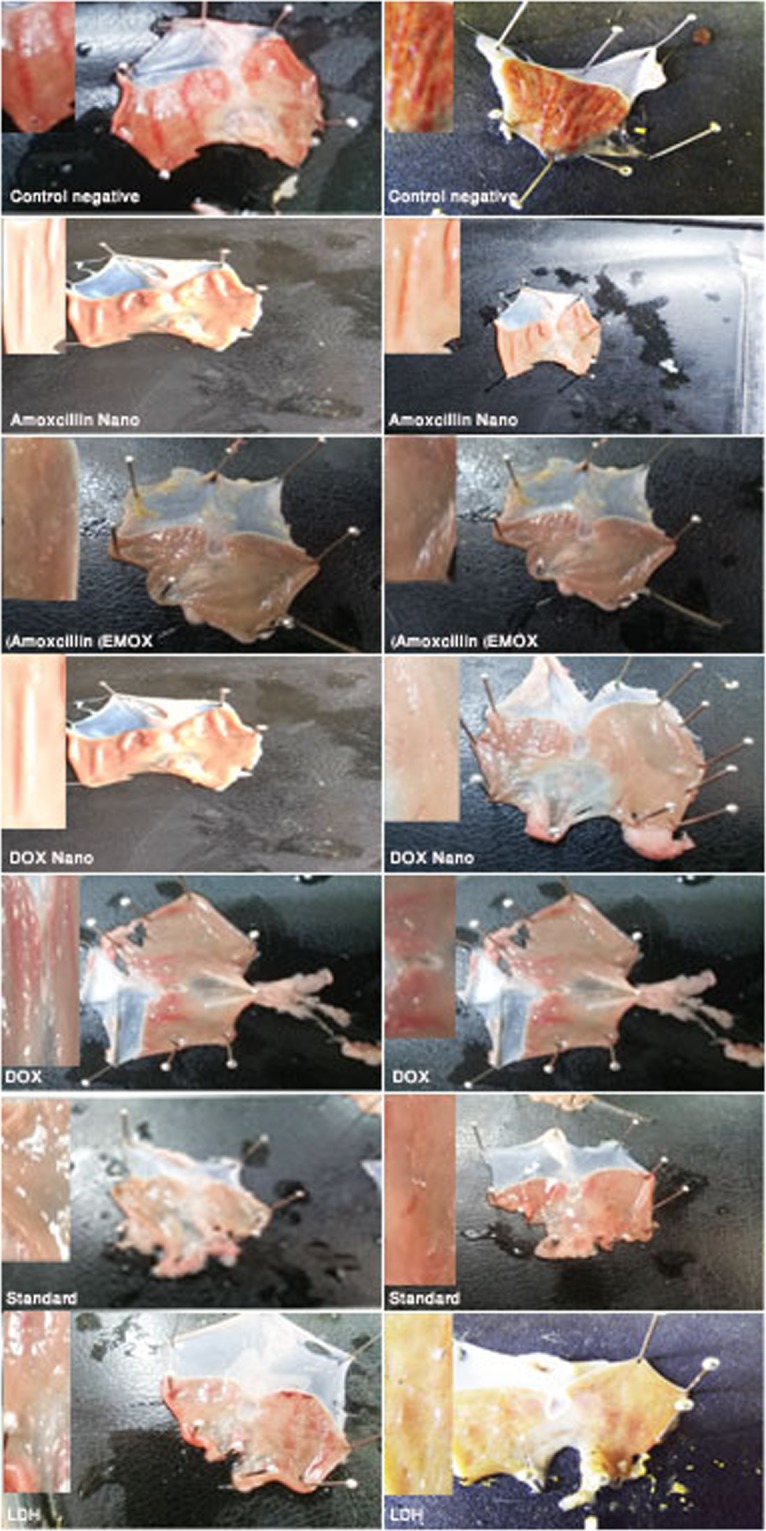
Anti-Ulcerogenic effect of G1 (CNT), G2 (AMOX/LDH), G3 (AMOX), G4 (DOX/LDH), G5 (DOX), G6 (standard), and G7 (Mg AL) in rats. (n = 6).

Oral administration of biotechnological Absolute Ethanol 98.9% to 24-hour fasting rats induced higher ulcer index in a control non-treated group *(P* < *0*.*00)* compared to other treated groups. Significant decrease in the ulcer index was observed in groups pre-treated with DOX/LDH (*p* < *0*.*000)* and AMOX/LDH *(p* < *0*.*000)* with higher protection rate when compared to a control non-treated group. A Standard group administered with Ranitidine^®^ also showed significant decrease (*p* < *0*.*000)* in the ulcer index compared to a control group. Rats treated with AMOX/LDH (G2) *(p* > *0*.*05)*, DOX/LDH (G4) *(p* > *0*.*05)*, AMOX (G3) *(p* > *0*.*05)* and standard group (G6) *(p* > *0*.*05)*, on the other hand, showed non-significant variation in comparison with each other, however it revealed significant difference when compared to DOX (G5) *(p* < *0*.*05)* and Mg-Al (G7) *(p* < *0*.*05)*. These two groups G5 *(p* > *0*.*05)* and G7 *(p* > *0*.*05)* showed non-significant variation between each other but significantly differed from the non-treated control group. The maximum percentage of gastric protection was observed in the DOX/LDH, AMOX/LDH and standard group in comparison to other groups (Table 5) and (Fig. 6).

**Table 5:** Anti-Ulcerogenic effect of DOX/LDH and AMOX/LDH Nano composite in rats. (n = 6).

Group	Dose mg/kg. b.wt.	Mean ± Standard error (SE)		percentage of protection
		Ulcer number	Ulcer index	
G1 Control	—	45.66 ± 1.41^a^	131.66 ± 2.1^a^	0
G2 AMOX/LDH	40	9.00 ± 0.5^cb^	35 ± 2.8	73.41
G3 AMOX	40	7.00 ± 0.5	36 ± 0.5	72.65
G4 DOX/LDH	5	5.66 ± 1.2	16.33 ± 8.8	87.59
G5 DOX	5	20.6 ± 2.3^b^	75 ± 2.8^c^	43.04
G6 Standard (Ranitidine®)	50	5.00 ± 1.7	5 ± 1.1	96.20
G7 Mg-Al	5	25.00 ± 2.8^b^	91.6 ± 0.9^bc^	30.42

Means within the same column having the same or no manuscript denote non-significant variation *(P* < *0*.*05*).

**Figure 7:**
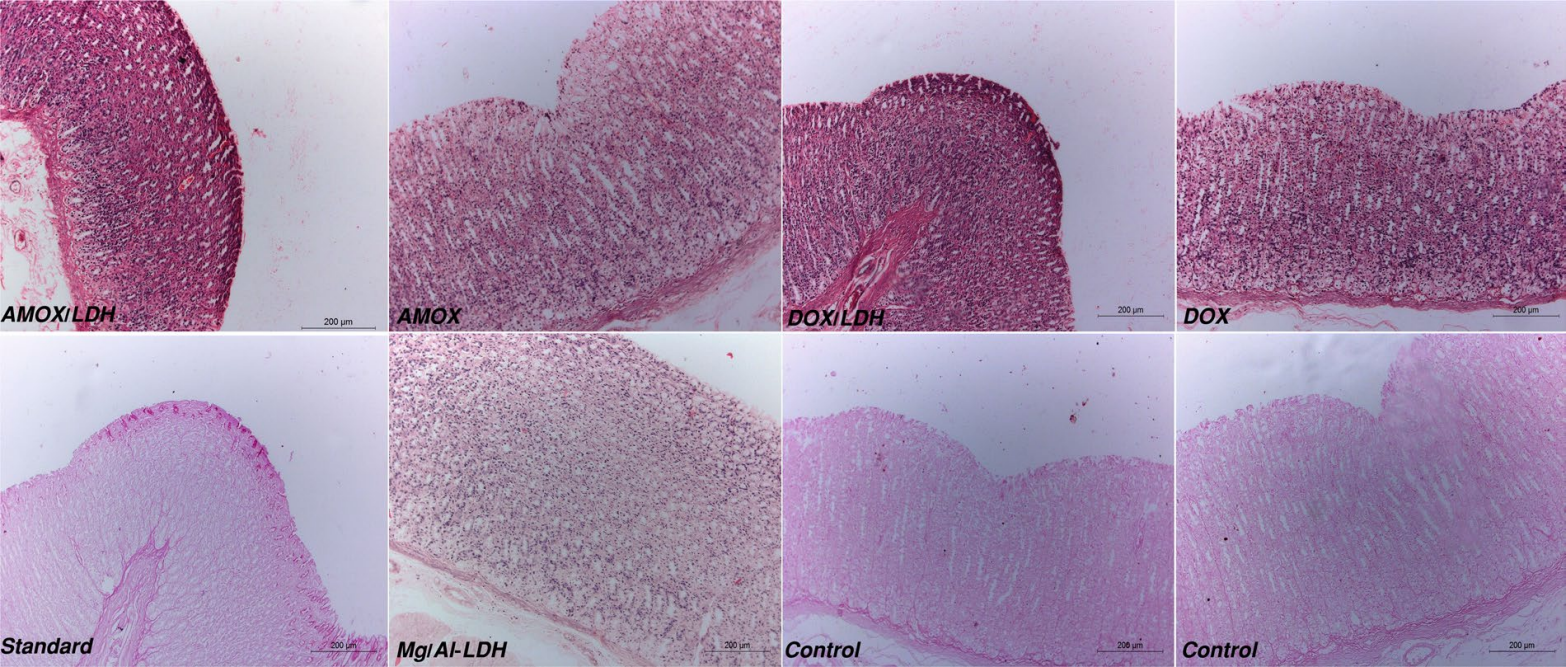
Histopathological studies of the different treatment on stomach’s Ulcer in rats. (n = 3). Both AMOX/LDH and DOX/LDH revealed normal gastric structure with complete gastric protection from ulcer. Amoxicillin, Doxycycline and Control groups showed severe mucosal damage with high congestion. Standard and Mg/Al showed good mucosal protection with normal structure.

Histopathological investigation into the different treatments of the stomach revealed good gastric and mucosal protection with normal structure in the DOX/LDH, AMOX/LDH, Standard and Mg/Al, while deep gastric ulcer was observed in DOX, AMOX. and the Control Groups (Fig. 7).

Rapid clearance and metabolism, instability in the biological fluids, high dosing, systemic adverse effects and limitation to reach to the site of action are the major drawbacks for the antimicrobial use^37^. Drug Delivery system using nanotechnology is now a very important model for increasing drugs therapeutic efficacy, active or passive targeting, controlled release and eliminating the systemic drug adverse effects^38^. To the best of our knowledge, although both doxycycline and amoxicillin are commonly-used broad spectrum anti-microbial drugs for the treatment of multiple diseases in humans and animals. No previous studies were conducted concerning the loading of such drugs into Layered Double Hydroxide (LDH) nanocomposite for exploring novel applications. Similarly no previous data referred to wound healing activity or ulcer treatment of DOX/LDH or AMOX/LDH Nanocomposites. The plan of this study included two main targets, as follows: Incorporation of doxycycline and amoxicillin into Mg/Al LDH in the form of antimicrobial nanocomposites, as well as investigating their ability for wound healing and ulcer treatment *in vivo* besides screening their acute toxicity.

Layered double hydroxide (LDH) demonstrated a great significance as a novel model for drug delivery system in the branch of pharmacology because of their safety and low toxicity^38^.

In the acute toxicity study, LD_50_ was used to determine the acute toxicity of both DOX/LDH and AMOX/LDH nanocomposites. In DOX/LDH nanocomposites, mice showed toxic symptoms and mortality starting at 600 mg/kg b.wt., and maximum mortality (LD_100_) was reached at 1600 mg/kg b.wt. By contrast, the mortality of AMOX/LDH nanocomposites started at 1000 mg/kg b.wt., with the maximum mortality (LD_100_) reached at 1500 mg/kg b.wt. The LD_50_ of DOX/LDH was 1100, whereas that of AMOX/LDH was 1210; thus, both were considered safe drugs. Mice treated with either DOX/LDH or AMOX/LDH showed no toxic symptoms or mortality after treatment with normal dosing, which indicates the drugs’ safety for use. Drugs or materials with an LD_50_ value of 1000 mg/kg b.wt. |are considered safe or of low toxicity after oral administration^39^; therefore, both DOX/LDH or AMOX/LDH are considered safe drugs. Similarly, LDHs based on magnesium or aluminium are considered the safest nanomaterials for drug delivery and the least toxic compared to other LDH types^40^. Therefore, LDH is widely used as a nanomaterial carrier for drug delivery systems or other substances, such as proteins or genes^41^.

Nanotechnology displays great appropriateness in improving wound healing treatments. The Nano-Meter scale opened the way for the evolution of novel materials for use in highly advanced medical technologies and revival targeting efficiency of the multifunctional Nano carriers. Incorporation of small molecules of drugs into nanoparticles or layers might modulate their safety, bioavailability and efficiency^42^. The pharmacokinetics and Pharmacodynamics of drugs are greatly affected by the Nano carrier size^43^.

Wound healing still remains a challenging clinical problem for which efficient wound handling and care is necessary. Moreover, the effective repair of wound and tissues is still a major healthcare and biomedical challenge in the 21^st^ century. Infected or chronic wounds often lead to loss of life through loss of the ability to perform the desired function and the increased pain severity in addition to being a load on healthcare system resources^1^, so searching for methods or drugs that can help in accelerating the wound healing process and shortening the period of wounds complete recovery would disclose a therapy of great importance. Percentage of healing activity and closure size of wound throughout 22 days were used as a marker for wound healing activity besides the histopathological findings. After Topical Application, DOX/LDH Nanocomposites showed a rapid and complete wound healing process in a shorter time, as with AMOX/LDH nanocomposites, and better than free drugs alone and non-treated group. In the same way, DOX, AMOX, LDH (Mg/Al) and standard group showed good healing activity but in longer time periods than DOX/LDH and AMOX/LDH nanocomposites ointment. The good healing activity of DOX/LDH and AMOX/LDH nanocomposites may be attributed to the good wound penetration power of the nanomaterial carrying the anti-microbial agents. The main cause of the un-healed wounds is the bacterial infections^44^, thus Doxycycline and Amoxicillin broad-spectrum Antibiotics against gram-positive and gram-negative bacteria are used for loading on LDH Nanomaterial. *Staphylococcus* is one of the most common bacterial pathogens responsible for the majority of wound infections and is considered as one of the major causes of hospital-acquired infections. Weak Antimicrobial agents or that of poor invasion ability can lead to bacteremia, sepsis and/or toxic shock syndrome^45^. The good penetration power of both Doxycycline and Amoxicillin to the wound area through the Nano materials prevents the occurrence of any bacterial infection and helps in accelerating the wound recovery process with complete fur formation and without the presence of scabs.

Histopathological findings indicated that the Amoxicillin and Doxycycline nanocomposites showed rapid contraction, which is very important for fast wound closure, especially for animals with lose skins (mouse, rat). Re-epithelialization was a common stage for all animals during the wound healing process and contact epithelial surface^46^.

Nanoparticles enhanced the activity of the carrier materials such as drugs due to their large surface-to-volume ratio. Besides, the efficacy of the antimicrobial activity of the doxycycline and amoxicillin increases when using LDH Nano layers^47^. Both Magnesium and Aluminum ions of LDH have antimicrobial activity and act as free radical scavengers for ROS (Reactive Oxygen Species). Oxidative stress, in addition, leads to an increased ROS production and also postpones the cellular processes involved in wound healing. Consequently Mg/Al helps in gaining a good healing activity in a shorter time^48^.

The main reason for the mucosal un-healed wound is the bacterial infection leading to gastrointestinal ulcer (GIU), which is mainly caused by *Helicobacter pyloriis* bacteria in adults and children worldwide. Failure of treatment with sufficient antibiotics concentration is due to that *H*.*pyloriis* lives beneath the gastric mucous lining supporting the gastric epithelium^5^. Nanoparticles loaded with antibiotics had shown effective treatment of gastrointestinal infections as their small size particles adhere efficiently on the gastric mucosa and act well against the bacteria living there^7^. On that basis, both DOX/LDH and AMOX/LDH nanocomposites treated groups showed a significant decrease in the ulcer index or in the severity of the induced ulcer after 7 days of treatment. The decrease in ulcer induction might be attributed to the perfect adhesion of the Mg/Al-LDH nanomaterial due to their small size which later allows controlled antibiotic release. The possible mechanism of gastrointestinal protection form ulcer or wound through the ulcerated tissue especially in stomachs producing large amounts of mucous which favors adhesion of small size particles due to their small mass. In addition, gastric inflammation increases the mucous production and fine particles adhere more perfectly^49^. At the inflammation site, immune cells, such as macrophages, engulf small particles and consequently load the antibiotics that release and produce localized effects^50^.

In addition, both Doxycycline and Amoxicillin Antibiotics treated groups also showed a significant decrease in the number of ulcers. This decrease might be attributed to their antibacterial activity in wound or ulcer treatment^51^. On the same ground, Mg/Al LDH showed a decrease in the ulcers numbers when compared to control non-treated rats. This decrease or that gastric protection was caused by the protective layer formed by the Mg/Al LDH which was commonly used as an anti-acid^52^. Ulcer diseases treated with LDH Nano-carrier in the form of oral anti-acid and anti-pepsin confirmed the safety and biocompatibility of this Nanos delivery system, which is also confirmed by the histopathological findings.

To the best of our knowledge, there were no reported previous studies dealing with the efficacy of the antibiotics mentioned herein above for wound or ulcer treatment, however there exists multiple studies showing the importance of topical antibiotic administration in the healing rate and prevention of infection. Nowadays topical antimicrobial therapy for ulcers or wounds has become more firmly established due to its importance with regard to the healing rate. Healing time is the most important endpoint in wound management^53^. Some researchers have noted a healing rate of 83% in 30 weeks^54^ while others have reported that 62/90 (69%) of venous leg ulcers had been healed within 12 weeks. Moffatt *et al*.^55^ found that 70% of venous ulcers healed after 48 weeks of treatment; the authors also noted that much of the evidence on healing rates is drawn from the results of randomized controlled trials. These trials typically achieve 24-week healing rates in excess of 60% for patients but may not reflect the complex issues faced in clinical practice. Due to these studies, the conversion of antibiotics to the nano form will have an important effect on the healing rate within a shorter time, as in our study.

The main idea of this study revolves around the topical application of antibiotics with high penetration power and prolonged release rate through loading it on Nanomaterials. Applying such antibiotics topically on wounds prevents from the infections that delay the healing rate, so that the wounds and ulcers’ curing process takes a shorter time. Consequently, we found that adding antibiotics accelerates the healing process and lessens the healing time through preventing infections.

The advantages of topical therapy include the ability to deliver a high local concentration with small doses of the agent, even in patients with limb ischaemia, to avoid the first-pass effect in the gastrointestinal tract, as well as reducing the risks of systemic side effects. Topical formulations achieve very high local concentrations^56^. In addition, the eradication or reduction of microorganisms in the wound alone is not a sufficient endpoint for drug efficacy^57^ but also depends mainly on their penetration power and its efficacy. Finally, no clinical data support the use of topical antibiotic treatment for the prevention of wound infection recurrences. All open wounds are colonized by microorganisms, which generally affect the healing process. However, if the colonization develops into local infection, which consequently becomes systemic, the result can be life-threatening. Accordingly, wound care not only comprises cleansing, debridement and management of the underlying aetiology but also takes measures for preventing the probability of colonized wounds becoming locally, or even systemically, infected^58^. It has been demonstrated that local antibiotic treatments play a role in promoting healing, although treatment of the underlying aetiology remains a crucial element^59^. Local factors that can delay healing include the number of bacteria present on the wound surface^60^. Moreover, both magnesium and aluminium hydroxide essentially act as antacid agents through the gastric coating^61^. Besides, Amoxicillin plays a role against *Helicobacter bacteria* and exhibits the broad-spectrum activity of Doxycycline. All these factors are the main reasons for selecting such materials for evaluating their activity in wound and ulcer healing.

The use of antibiotics with any injury (Wound or Ulcer) is the main treatment for rapid healing in a shorter time. This is done through Infection Prevention which is the main cause for delaying the healing of the wound or ulcer. As infection is a common reason for poor wound healing. Accordingly, a significant reduction in the number of bacteria is important for the management of either acute or chronic wounds especially the bacterial growth inhibited inside the induced or opened wounds by lowering the pH values^62^. Wound surface induction lowers the pH of the infected surfaces and establishes an unsuitable environment for the growth and multiplication of the bacteria. In addition, the pH of a wound can additionally influence the effectiveness of antibiotics and antiseptics^63^. The pH may modulate the effectiveness; and success of antibiotic performance potentially altering the metabolic state of bacteria^23^, allowing bacterial growth and acquired resistance to occur. For example, multiple antibiotics and silver antiseptic’s effectiveness is reduced in acidic environments which is also the favorable media in our study^64^.

The main role of antibiotics in wound healing is inhibiting the growth of drug-resistant bacteria and promoting healing in a rodent wound model^65^. Both amoxicillin and doxycycline fulfil these roles as broad-spectrum and bactericidal antibiotics that help prevent bacterial growth and help ulcers and wounds heal within a short period. Thus, the nanoparticles implemented in this study exhibited much higher antibacterial potency against Gram-negative and Gram-positive bacteria as they increased the penetration power into the bacterial cell wall or membrane. The enhanced inhibitory effect compared with that of the normal antibiotic alone was due to the ability of the designed nanoparticles to disintegrate the bacterial membrane. These nanoparticles accelerated the healing of ulcers and wounds by regulating inflammation and angiogenesis. In addition, they allowed for an increase in the skin absorptivity of the nanoscale mixture.

The majority of these nanoparticle carriers, such as LDH, have been used for the delivery of therapeutic agents with antibacterial properties, and they have been explored as Nano carriers of wound-healing drugs, chosen based on wound needs^13^. These Nano carriers help drug delivery, as they promote cell growth, accelerate cutaneous wound healing, and reduce scar formation^66^.

In particular, *H*. *pylori* lives deep within the gastric mucus, and prolonged local drug application is needed for sufficient diffusion to the bacteria^67^. No single antibiotic is effective in eradicating *H*. *pylori* when administered *in vivo*. The treatment of such infectious disease in peptic ulcers usually requires a triple therapy that includes antibiotic, antibacterial, and proton pump inhibitors. The failure of a single antibiotic therapy could be due to poor stability of the drug in the acidic pH of the stomach, poor permeability of antibiotics across the mucus layer, or the availability of sub-therapeutic antibiotic concentrations at the infection site after oral administration in a conventional dosage form^68^. Therefore, synthesis of antibiotics in nano form helps the antibiotics penetrate into the deep mucous layer and improves bactericidal efficacy against *H*. *pylori*.

To improve the efficiency of such therapeutic modalities, site-specific antibiotic drug delivery systems have been developed for localized treatment of *H*. *pylori* infections of the stomach. Some attempts have been made to develop localized antibiotic delivery systems in the acidic environment of the stomach. For this reason, we also chose pH 4 as an acidic medium to improve drug stability. Amoxicillin (“-amino-hydroxy-benzyl penicillin) is a semi-synthetic, orally absorbed and broad-spectrum antibiotic. The antibiotic is widely used in the standard eradication treatment of gastric *H*. *pylori* infection, and using nanosynthesis will help achieve greater drug penetration and therefore higher bactericidal efficiency^27^.

Ideal candidates for the dressing of infectious wounds should sufficiently deliver high concentrations of antibiotics to the wound site; mimic the structure and biological function of native extracellular matrix proteins, which provide support and regulate cellular activities; maintain the normal state of differentiation within the cellular compartment; be biocompatible and/or biodegradable; and have no adverse effects on the surrounding tissue^69^. Based on these factors, the main objective and the principle step for wound healing is to prevent infection through the use of broad-spectrum and bactericidal antibiotics in particular, such as those employed in our research study. In addition, the loading of broad-spectrum and bactericidal antibiotics on nanomaterials helps the drugs achieve greater penetration power inside the wound or ulcer induced. Internationally, antibiotic-resistant bacteria and multidrug-resistant bacteria are increasing as a clinical problem; these bacteria have been found in isolates from a substantial proportion of patients with pressure ulcers and even in community settings^51^.

Another mechanism by which these results were obtained, other than the antimicrobial efficacy achieved, is through the LDH itself, which helps improve antibiotic efficacy, as layered hydroxides have been considered unique Nano-carriers for efficient cellular delivery of drugs. This ability is due to the excellent features of LDHs, especially the unique surface modification achieved due to their favourable ion-exchange properties. In addition, the positively charged layers of layered hydroxides significantly improve the efficient cellular delivery of drugs. The negatively charged membrane of cells prevents drugs with a negative charge from entering. By incorporating anionic drugs into the layers of layered hydroxides, these drug-nanohybrids with positively charged surfaces are able to properly enter the cell and enhance the cellular delivery of drugs^51,70,71^. By increasing the particle size of LDH layers or sheets, the cellular uptake of LDH is decreased, but interestingly, it has been observed that the cellular uptake of all sizes of layered hydroxides is completed within 15 min through the retention time mechanism^72–74^. LDHs have an added advantage in delivering negatively charged drugs because they bear a net positive charge on their surface that facilitates adsorption as well as internalization through the negatively charged biological membranes without the need for any additional post-modification/functionalization, as in the case of other materials. Thus, many researchers have conjugated a variety of negatively charged cytotoxic drugs into the interspaces of LDHs and successfully delivered them into cells with controlled release properties^75,76^.

In conclusion, the doxycycline-amoxicillin nanocomposites developed in this study (i.e., Mg/Al LDH) hold great prospects for treating wounds (ointments) within a shorter time and for ulcer prevention (oral dosing). Conversion of antimicrobial drugs, especially antibiotics, to nanoscale drugs enables them to acquire new properties, such as wound healing activity and ulcer prevention, through a much better route than that of the standard drugs used. This ability is realized through the prevention of infection via higher penetration power and high efficacy against pathogenic microorganisms that infect open wounds or mucosal injuries and cause delays in healing rates of infection and life-threatening problems.

## Methods

### Materials

The salts of aluminium [Al (NO_3_)_3_.9H_2_O] and magnesium [Mg (NO_3_)_2_.6H_2_O] were purchased from Oxford Refractory Reagent, India. Sodium hydroxide (NaOH) and hydrochloric acid (HCl) were obtained from the Biochemical Laboratory, Egypt. Doxycycline and amoxicillin of 100% purity were obtained in a water-soluble form from Pharma Swede Pharmaceutical Company, Egypt. Ranitidine hydrochloride (Rantidine), was obtained from Medical Union Pharmaceutical Company, A.R.E, in the form of 150 mg tablets. Tubes of sodium fucidate ointment (Fucidin) were obtained from MINAPHARM Company, Egypt, with each tube contains 20 g of ointment. Medical biotechnology absolute ethyl alcohol (98.9%) used for ulcer induction was obtained from Biochemical Laboratory.

### Synthesis of LDH, doxycycline/LDH and amoxicillin/LDH

Mg (NO_3_)_2_.6H_2_O (0.045 mol) and Al (NO_3_)_3_.9H_2_O (0.015 mol) (i.e., Mg:Al molar ratio of 3:1) were dissolved in 100 mL distilled water. Sodium hydroxide (NaOH) was added dropwise until complete precipitation at pH 10.0. The precipitate suspension was stirred for 20 h at 65 °C, filtered, washed several times with double-distilled water and finally dried at 40 °C.

Doxycycline-LDH was synthesized by repeating the same procedures and adding 0.005 mol of doxycycline to the medium before precipitation. The doxycycline-LDH-precipitate suspension was stirred at room temperature for 20 h, filtered, washed, and dried at 40 °C. Amoxicillin-LDH was synthesized by adding a solution of amoxicillin (1 g of amoxicillin being dissolved in 50 ml water, with the pH adjusted to pH 7 by NaOH) to Mg: AL LDH for 24 h at room temperature. The precipitate was filtered, washed and dried at 40 °C. All steps are shown in Scheme (1) (Supplementary Material 1).

### Characterization

The crystal structure and crystallinity of the materials were characterized by XRD. The vibrations of the materials chemical bonds were examined by Fourier transform infrared spectroscopy (FT-IR, Bruker Vertex 70). High-resolution transmission electron microscopy (HRTEM, JEOL-JEM 2100) was used to characterize the microstructure of all nanocomposites. The morphology and elemental analysis (EDAX) of Mg-Al/LDH was characterized by a field emission scanning electron microscope (FESEM, Quanta FEG 250) to confirm the ratio of Mg-Al LDH (3 Mg:1 Al), as shown in Scheme (1). The particle sizes and zeta potentials were studied (experimentally optimized S1 (Supplementary Fig. 2) by a Malvern instrument (Malvern Instruments Ltd). The method of sample preparation is mentioned in our previous work^14–17^.

### Experimental Animals

Rats and mice were obtained from the lab animal unit, and rats were obtained from the Lab Animal Unit, Department of Physiology, Faculty of Veterinary Medicine, Beni-Suef University, Egypt. They were kept in standard laboratory conditions at 22 ± 3 °C, 60 ± 5% humidity and a 12-h light/dark cycle. Animal handling methods, including weighing and gavage procedures, were carried out in accordance with and were approved by the Institutional Animal Care and Use Committee, Faculty of Veterinary Medicine, Beni-Suef University (Protocol of Animal Rights for Laboratory Experiments). A standard 12-h light and dark protocol was applied throughout the experiment. Mature mice weighing 20–40 g b.wt. of both sexes were used for the determination of the LD_50_ values of the doxycycline-LDH and amoxicillin-LDH nanocomposites for toxicological studies. Mature albino rats of both sexes, with body weights of 150–250 g, were reared on a standard diet daily with continuously available water for 24 h. The experimental study was carried out after 7days of acclimatization of the rats with a standard protocol of 12 h of light and dark and for each group in its respective metal cage.

### Experimental Groups and Medications

Eighty four (84) adult Albino Rats of both sexes (Average body weight 150–250 gm, average age 3–4 months), were used in this study for both wound healing and ulcer prevention experiment. Rats were divided into 7 equal groups each of six rats for each experiment. As For Ulcers, rats in group one (G1) were considered as control-negative (CNT) and received orally 0.5 ml of D.W. Group two (G2) received Amoxicillin-LDH Nano composite (AMOX/LDH) at dose of 40 mg/kg B.wt., Group three (G3) administered Amoxicillin (AMOX) drug alone also at dose of 40 mg/kg B.wt., Group four (G4) was treated with Doxycycline-LDH Nano composite (DOX/LDH) at dose of 5 mg/Kg B.wt., and Group five (G5) received Doxycycline alone at the same dose, Rats in group six (G6) were considered a Standard Group that received Ranitidine (50 mg/kg B.wt.) for ulcer study while using Fucidine ointment for wound healing. Rats in group seven (G7) were treated with LDH material by a dose of 5 mg/kg B.wt.

All groups including the control-group were treated orally with stomach tube gavage daily for 7 continuous days for ulcer study. All drugs were made in the form of an ointment 10% based on Vaseline and were applied on the wound area for continuous 18 days.

### Toxicological Studies

For safe drugs use, an acute toxicological study LD_50_ was carried out for Doxycycline-LDH and Amoxicillin-LDH Nanocomposites. A Total number of 110 mice, fifty-five mice of which were used for each tested Nano-drug. The mice were used for LD_50_ estimation according to^77^ with some modifications. LD_50_ investigation was involved in three phases. Mice were divided into three groups (Each of five mice) and the tested drugs were administered orally in upgrading doses in DOX/LDH as follows: We started with 200, 400 and 500 mg/kg b.wt. then in the second stage another three groups were administered 600, 700, 800, and 1000 mg/kg b.wt then finally, another three groups according to the observation, were administered 1200, 1400 and 1600 mg/kg.b.wt. Another group was left as a Control-group and was given diluent only.

In AMOX/LDH group, we started with 800, 900 and 1000 at first then increasing the upgrading doses as 1000, 1100, 1200, 1300 and finally the doses were 1300, 1400 and 1500 in addition to the control-negative group. The toxic symptoms, mortality rate and post-mortem findings in each group were recorded 24 hours post administration. LD_50_ of tested drug was calculated according to the following formula:

**LD**_**50**_ was calculated according to the following formula (Equation 1):

1 $${{\rm{LD}}}_{50}={\rm{DM}}-\frac{\sum ({\rm{A}}\times {\rm{B}})}{{\rm{N}}}$$

where:

**DM**: The dose caused 100% mortality. Larger doses which killed all animals.

**A:** Constant factor between two successive doses, Dose difference between 2 successive groups

**B:** Average of dead animals between two successive groups.

**N:** Number of animals in each group,

**Σ**: **S**ummation of multiplying A and B.

### Estimation of Wound healing activity

Surgical incised wound of (10 × 10 mm) size had been made on the back of the rats present in ventral posture after induction of anaesthesia through intraperitoneal injection of ketamine 5% (90 mg/kg B.wt.) and xylazine hydrochloride 2% (5 mg/kg B.wt.). Fur of each rat was aseptically removed from the back and an area of 1 × 1 cm (10 × 10 mm) was labelled with a marker before starting the wound incision. The wound healing activity was determined according to the method stated^78^.

Rats were divided according to treatment into seven (7) groups, while G1 control negative (CNT) was applied with Vaseline only, G2, AMOX/LDH Nanocomposite ointment, G3, AMOX ointment, G4, DOX/LDH Nano composite ointment, G5, DOX ointment, G6, Fucidine ointment and G7, LDH ointment. All treatments were made in the form of ointment based on Vaseline of 10% concentration which was applied topically on the wound area once daily after 2 hour post-operation until the wound was completely healed. All animals kept under good hygienic and controlled conditions. All rats were monitored daily and any wound fluid or evidence of infection or other abnormalities were noted. Wound contraction percentage and wound closure time were used to assess wound-healing activity. The wound size was computed on days 4, 8, 12 and 16 days post-operation. The wound healing percentage was calculated by the Walker formula^79^ (Equation 2).

2 $${\rm{Percentage}}\,{\rm{of}}\,{\rm{wound}}\,{\rm{size}}=\frac{{\rm{Wound}}\,{\rm{area}}\,{\rm{on}}\,{\rm{day}}\,{\rm{X}}}{\,\mathrm{Wound}\,{\rm{area}}\,{\rm{on}}\,{\rm{day}}\,{\rm{zero}}}\times 100$$

### Anti-Ulcerogenic effect of DOX/LDH and AMOX/LDH Nanocomposite

Induction of gastric ulcer induced by oral administration of biotechnological absolute ethanol produces gastric lesion in rats as recorded^80^ with slight modification and the method of ulcer calculation^81,82^.

Rats were divided, in relation to the treatment, into G1 (Control-Negative) administered daily 0.5 ml D.W., G2, a dose of 40 mg/kg B.wt.AMOX/LDH, G3, A dose of 40 mg/kg B.wt AMOX. G4, A dose of 5 mg/Kg B.wt DOX/LDH. G5, A dose of 5 mg/Kg B.wt DOX. G6, standard group revived Ranitidine 50 mg/kg B.wt. and G7, LDH with a dose of 5 mg/Kg B.wt.

All the treatments were administered once daily for 6 days and at the 7^th^ day rats were starved for 24 h, except for water, until the last hour before the induction of ulcer. A dose of 5 ml/kg B.wt Absolute biotechnological ethanol 98.9% was administered to all rats, but one hour later all groups were given the last dose of treatment. One hour later after treatment, rats died by their anaesthesia with Ketamine mixed with xylazine with (0.1 ml/100 gm) then cervical dislocation. The abdominal cavity was incised to get the stomach, the stomach was opened along its greater curvature, rinsed with physiological saline solution and pinned flat on a cardboard to be exposed for gross lesions evaluation before impeding it in formalin 10% for histopathological studies.

By the help of illuminated magnifying lens (10x) the gastric mucosa was investigated for counting ulcer numbers^83^. Using transparent ruler the lesions were counted and measured along the greater diameter. At calculation each five haemorrhagic spots equal to 1 mm. The sum of the total length of long ulcers and haemorrhagic spots in each group of rats was divided by the number of animals to calculate the ulcer index (mm). The percentage of protection was calculated according to the method described by^84^ (equation 3).

3 $$ \% \,\mathrm{of}\,\mathrm{protection}=\frac{({\rm{ulcer}}\,{\rm{index}}\,{\rm{of}}\,{\rm{control}}-{\rm{ulcer}}\,{\rm{index}}\,{\rm{of}}\,{\rm{treated}})}{{\rm{ulcer}}\,{\rm{index}}\,{\rm{of}}\,{\rm{control}}}\times 100$$

### Histopathological examination for Wound and Ulcer

A total of 28 rats (4 rat/group) were randomly selected and euthanized at the 16^th^ day of the experiment, parts from the healed area of the skin were obtained while in the Ulcer Study the stomach collected at the 7^th^ day and all samples were excised and fixed in neutral-buffered formalin 10% for histopathological study. The processed samples and the obtained sections (5 µm) were stained with haematoxylin and eosin (H&E) examined microscopically for the evaluation of the cellular or immunological infiltration or macrophages in the wound area, collagen or fibroblast percentage, and vascularization rate^85^.

### Statistical analysis

The results were expressed as mean ± standard error of mean (S.E.M.). Statistical significance was determined through a one-way analysis of variance (ANOVA) according to^86^, and then it was followed by Tukey’s post-hoc test for multiple comparisons using SPSS (version 20.0) software (IBM SPSS Statistic 20.0, Armonk, NY, USA). The *P* values less than 0.05 were considered statistically significant.

## Supplementary information

Supplementary Info File
