# Supplementary material for: New Approach in Ulcer Prevention and Wound Healing Treatment using Doxycycline and Amoxicillin/LDH Nanocomposites
Source: Sci Rep. 2019 Apr 23;9:6418. doi: 10.1038/s41598-019-42842-2 (PMC6478882; doi:10.1038/s41598-019-42842-2)
Supplement: Supplementary file 1 — Supplementary Info File [file 41598_2019_42842_MOESM1_ESM.pdf]

New Approach in Ulcer Prevention and Wound Healing Treatment using Doxycycline and Amoxicillin/LDH Nanocomposites

Fatma I. Abo El-Ela[1*], Ahmed A. Farghali[2], Rehab K. Mahmoud[3], Nada A. Mohamed[4], S. A. Abdel Moaty[5]

[2], Materials Science and Nanotechnology Department, Faculty of Postgraduate Studies for Sciences, Beni-Suef University, Egypt, E-mail: d_farghali@yahoo.com

[3], Department of Chemistry, Faculty of Science, Beni-Suef University, Beni-Suef, Egypt, E-mail: radwaraft@yahoo.com

[4], Department of Chemistry, Faculty of Science, Beni-Suef University, Beni-Suef, Egypt, E-mail: nadaabdelnaby@yahoo.com8

[5],Materials Science Lab, Department of Chemistry, Faculty of Science, Beni-Suef University, Beni-Suef, Egypt, E-mail: dr.samahchemy2010@yahoo.com

Corresponding author:

Fatma I. Abo El-Ela[1], Lecturer of Pharmacology, Department of Pharmacology, Faculty of Veterinary Medicine, Beni-Suef University,

e-mail: fa.pharma@yahoo.com, fatma.aboel3la@vet.bsu.edu.eg

Tel: +201152460404, Fax: +20822327982. Beni-Suef 62511, Egypt.

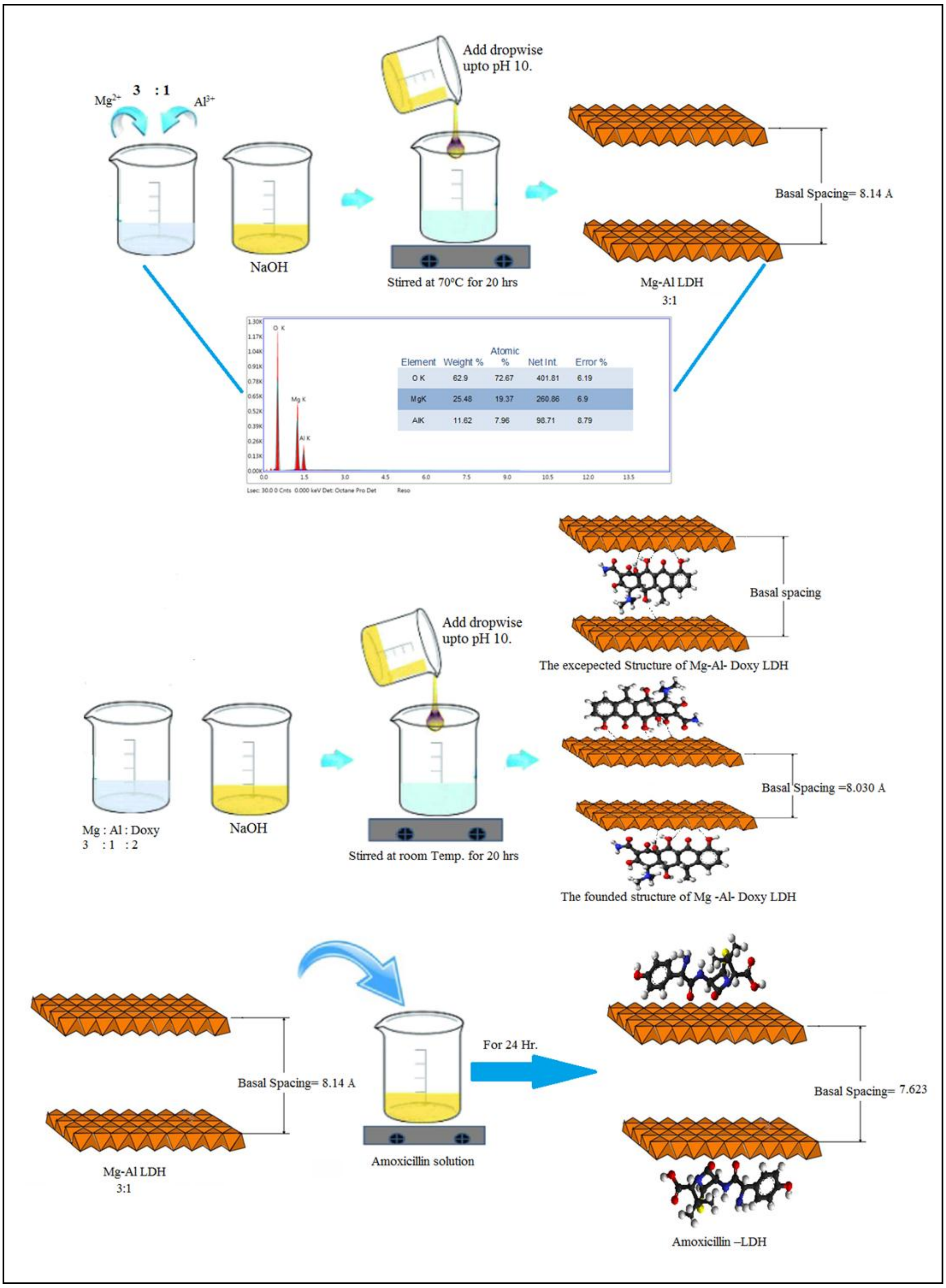

Scheme 1. Diagram shows the preparation steps of LDH, Doxycycline–LDH & Amoxicillin–LDH nanocomposite.

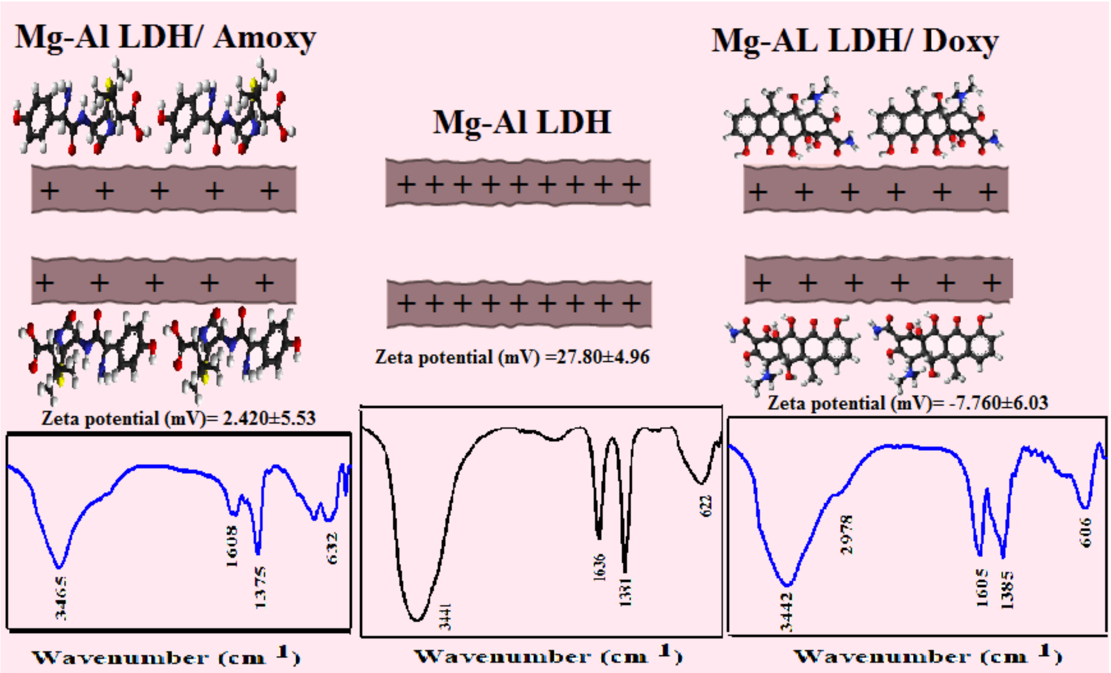

Supplementary figure
